# Comparison of breast cancer surrogate subtyping using a closed-system RT-qPCR breast cancer assay and immunohistochemistry on 100 core needle biopsies with matching surgical specimens

**DOI:** 10.1186/s12885-021-08171-2

**Published:** 2021-04-21

**Authors:** Slavica Janeva, Toshima Z. Parris, Salmir Nasic, Shahin De Lara, Karolina Larsson, Riccardo A. Audisio, Roger Olofsson Bagge, Anikó Kovács

**Affiliations:** 1 Sahlgrenska Breast Center, Department of Surgery, Sahlgrenska University Hospital, Region Västra Götaland, Gothenburg, Sweden; 2 Institute of Biomedicine, Sahlgrenska Academy, University of Gothenburg, Gothenburg, Sweden; 3 Institute of Clinical Sciences, Department of Oncology, Sahlgrenska Center for Cancer Research, Sahlgrenska Academy, University of Gothenburg, Gothenburg, Sweden; 4 Research and Development Centre, Skaraborg Hospital, Skövde, Sweden; 5 Department of Clinical Pathology, Sahlgrenska University Hospital, Region Västra Götaland, Gothenburg, Sweden; 6 Department of Oncology, Sahlgrenska University Hospital, Region Västra Götaland, Gothenburg, Sweden; 7 Institute of Clinical Sciences, Department of Surgery, Sahlgrenska Academy, University of Gothenburg, Gothenburg, Sweden; 8 Wallenberg Centre for Molecular and Translational Medicine, University of Gothenburg, Gothenburg, Sweden

**Keywords:** Surrogate subtyping, Breast cancer biomarker assays, Immunohistochemistry, PCR, mRNA, STRAT4

## Abstract

**Background:**

Routine clinical management of breast cancer (BC) currently depends on surrogate subtypes according to estrogen- (ER) and progesterone (PR) receptor, Ki-67, and HER2-status. However, there has been growing demand for reduced immunohistochemistry (IHC) turnaround times. The Xpert® Breast Cancer STRAT4* Assay (STRAT4)*, a standardized test for *ESR1*/*PGR*/*MKi67*/*ERBB2* mRNA biomarker assessment, takes less than 2 hours. Here, we compared the concordance between the STRAT4 and IHC/SISH, thereby evaluating the effect of method choice on surrogate subtype assessment and adjuvant treatment decisions.

**Methods:**

In total, 100 formalin-fixed paraffin-embedded core needle biopsy (CNB) samples and matching surgical specimens for 98 patients with primary invasive BC were evaluated using the STRAT4 assay. The concordance between STRAT4 and IHC was calculated for individual markers for the CNB and surgical specimens. In addition, we investigated whether changes in surrogate BC subtyping based on the STRAT4 results would change adjuvant treatment recommendations.

**Results:**

The overall percent agreement (OPA) between STRAT4 and IHC/SISH ranged between 76 and 99% for the different biomarkers. Concordance for all four biomarkers in the surgical specimens and CNBs was only 66 and 57%, respectively. In total, 74% of surgical specimens were concordant for subtype, regardless of the method used. IHC- and STRAT4-based subtyping for the surgical specimen were shown to be discordant for 25/98 patients and 18/25 patients would theoretically have been recommended a different adjuvant treatment, primarily receiving more chemotherapy and trastuzumab.

**Conclusions:**

A comparison of data from IHC/in situ hybridization and STRAT4 demonstrated that subsequent changes in surrogate subtyping for the surgical specimen may theoretically result in more adjuvant treatment given, primarily with chemotherapy and trastuzumab.

## Background

To provide the best pre- and postoperative treatment for a patient with early breast cancer, reliable pathology data about the biology of the tumor is needed [1–5]. Biomarker assessment of estrogen receptor (ER)-, progesterone receptor (PR)-, Ki-67, and HER2 status with immunohistochemistry (IHC) is used to classify breast cancer by intrinsic/surrogate subtypes according to international WHO guidelines: Luminal A-like, Luminal B-like (HER2- or HER2+), HER2+ (non-luminal) and Triple-negative [6]. Additional clinicopathological features (tumor grade and size, patient’s age and co-morbidities and axillary lymph node status) are then used for pre- or postoperative adjuvant treatment decisions.

Core needle biopsy (CNB) has become a well-established preoperative diagnostic method for breast lesions [6–9], especially for breast cancer patients being considered for neoadjuvant treatment [3]. International and national guidelines recommend the pathology-based assessment of ER, PR, HER2, Ki-67 [9] using immunohistochemistry, and good concordance has been shown for these biomarkers in matched CNB and surgical specimens [8, 10, 11]. However, IHC is frequently associated with interlaboratory variability due to differences in the choice of antibodies, manual or computer-assisted imaging scoring methods, as well as subjective interpretations by different pathologists [12–14]. Moreover, there is still considerable debate over optimal Ki-67 cutoffs to distinguish Luminal-like breast cancers [1, 15]. Subsequent in situ hybridization for HER2 testing (e.g. FISH, SISH) could take an additional 5 days before necessary information is obtained and optimal therapy planning can be conducted. Thus, there is a need to develop a fast, reliable, and reproducible method to standardize the assessment of ER, PR, HER2, and Ki-67.

A number of commercial gene signature assays (e.g. PAM50/Prosigna, Mammaprint/BluePrint) have been developed to classify breast cancer, thereby improving prognostication and treatment decision-making [16–18]. However, each molecular assay uses different technological platforms, gene signatures comprised of various numbers of transcripts with distinct biological functions, and benefits specific patient groups. The Xpert® Breast Cancer STRAT4 Assay (STRAT4)* is a cartridge-based, real-time quantitative polymerase chain reaction (RT-qPCR) assay with qualitative cut-off values for *ESR1*-, *PGR* -, *ERBB2*-, and *MKi67*- mRNA expression normalized to a reference gene (*CYFIP1*) using formalin-fixed paraffin-embedded (FFPE) invasive breast cancer tissue. RNA is automatically extracted, purified, and detected inside the cartridge using a tissue lysate prepared from a tumor-enriched region in the FFPE section, as identified by a pathologist. In comparison with traditional IHC, the total turnaround time for the STRAT4 assay is under 2 hours, including hands-on time. Previous studies have shown reproducible results and high concordance with IHC [19, 20].

The aim of the present study was to a) evaluate the concordance of the STRAT4 assay relative to standard-of-care IHC and SISH-test in matched breast cancer CNBs and surgical specimens, and b) investigate whether changes in STRAT4-based surrogate breast cancer subtyping of the surgical specimen would have had a theoretical effect on adjuvant treatment recommendations.

## Methods

### Patient samples

Formalin-fixed paraffin-embedded (FFPE) samples (i.e. preoperative CNBs and surgical tumor specimens) were retrieved from the Department of Clinical Pathology at Sahlgrenska University Hospital (Gothenburg, Sweden) for 98 consecutive patients with primary breast cancer diagnosed between January and May 2017. Two patients had bilateral breast cancer. In total, 100 matching samples were examined. The age of the FFPE samples was 18–22 months. Clinical data on adjuvant treatment based on IHC, axillary node status, tumor size, tumor grade, patient age at the time of diagnosis and co-morbidities were retrieved from electronic medical records. Patients with neoadjuvant treatment, tumor size less than 5 mm, multifocal/multicentric breast cancer, bifocal breast cancer and previous surgery for breast cancer were not included. Patients treated with neoadjuvant treatment were excluded to avoid the possible loss of available matching surgical samples for assessment due to partial or complete reduction of the tumor area following therapy response. Another exclusion criteria was FFPE specimens that were fixed in fixatives other than 10% Neutral Buffered Formalin (NBF), fixed in NBF for < 6 or > 72 h, or aged more than 4 weeks since initial preparation/fixation, but this was not relevant in this study. The clinicopathologic features of the 100 specimens are shown in Table 1.

**Table 1:** Clinicopathologic features for the 100 paired breast cancer cases (core biopsy and surgical specimen)

			Core biopsy	Surgical specimen
			*n* = 100	*n* = 100
Age at diagnosis Median (range)	61 years (35–93)			
Tumor histological subtype		IDC	76	76
		ILC	12	12
		Other	12	12
Tumor histological grade (Nottingham)		1	10	8
		2	71	61
		3	19	31
Tumor size (mm) Median (range)			¨	34.9 (8–159)
Axillary lymph node status		Positive	¨	47
		Negative	¨	51
		N/A^a^	¨	2
ER status ≥1% pos		Positive	87	87
		Negative	13	13
PR status ≥1% pos		Positive	78	81
		Negative	22	19
Ki-67 Median % staining (range)			15 (4–90)	17.5 (4–95)
HER2 status (HercepTest; SISH in case of score 2+ and 3+)		Positive	18	18
		Negative	82	82

^a^ axillary dissection was not performed

### Immunohistochemistry (IHC) and cutoffs for biomarker analysis in CNB and surgical specimen

ER, PR, HER2 and Ki-67 protein expression data (IHC) were retrospectively collected for both the CNB and surgical specimens from the Sympathy database (Sahlgrenska University Hospital, Department of Clinical Pathology) and no re-assessments were conducted. As the CNB sample is only a random sampling of the tumor, IHC assessment is routinely performed on both the CNB and the matching surgical specimen at our institution. Routine IHC staining protocols at the Department of Clinical Pathology used the following antibodies to assess ER, PR, HER2 and Ki-67 expression: rabbit anti-ERα (Agilent Dako IR084, clone EP1), mouse anti-PR (Agilent Dako IR068, clone 636), mouse anti-KI-67 (Agilent Dako IR626, clone MIB-1), and HercepTest (Agilent Dako SK001, clone poly), respectively. In brief, FFPE sections were pretreated using the Dako PTLink system (Agilent Dako) and processed on an automated Dako Autostainer platform, as previously described [21]. The Ventana dual SISH test was also performed, if necessary to resolve final HER2 status. International guidelines were used to determine biomarker status, where ER, PR, and Ki-67 were considered to be positive with ≥1%, ≥1%, and ≥ 20% immunostaining in neoplastic cells, respectively [1, 6]. HercepTest scored 2+ and 3+ was followed by SISH testing, and considered positive with confirmed HER2 amplification. SISH testing was not performed on the surgical specimen, if the matching CNB was HER2-amplified. New SISH tests were performed on the surgical specimen, for non HER2-amplified CNBs with HercepTest scored as 2+.

### STRAT4 analysis

STRAT4 analysis was performed in November 2018 and was limited to two operators (S.J. and S.D.L.), each blinded to the IHC status of individual biomarkers. For each patient, a single 4 μm or 5 μm FFPE section (adjacent to the section used for hematoxylin and eosin (H&E) staining, determined by a board-certified pathologist (A.K.)) was prepared for both the CNB and the matching surgical specimen. For some surgical specimens, macrodissection (tumor area outlined by the pathologist) was required to remove non-neoplastic tissue. The FFPE sections were first placed in a water bath at 40 °C, mounted onto positively charged FLEX IHC microscope slides (Agilent Dako) and dried overnight at room temperature. The FFPE section was then scraped from the slide and transferred to a 1.5 ml lysis tube. FFPE samples containing CNBs were sectioned and transferred directly to lysis tubes.

Tissue lysis was prepared by adding 1.2 ml Xpert® FFPE lysis kit reagent and 20 μl proteinase K (PK) to each sample, followed by incubation at 80 °C for 30 min. The tissue lysate was then transferred to a provided 5 ml sample vial and precipitated with 1.2 ml 95% ethanol. An aliquot containing 520 μl tissue lysate was transferred to the Xpert Breast Cancer STRAT4 cartridge, the cartridge lid was closed, and then the closed cartridge was placed into the GeneXpert instrument. Furthermore, nucleic acid purification, and RT-qPCR amplification and detection are fully automated in the GeneXpert instrument, with all the reagents required for the different stages preloaded in the cartridge. The GeneXpert instrument system simultaneously measured the cycle threshold (Ct) values in multiplex for the reference gene (*CYFIP1*) and target genes (E*SR1*, *PGR*, *MKi67*, and *ERBB2)*, and then calculated the delta cycle threshold (dCt) values (dCt = reference gene Ct minus target gene Ct) for each marker. The *CYFIP1* Ct and individual dCt values for each marker were then compared to pre-specified Ct and dCt cutoffs to classify *ESR1*, *PGR*, *MKi67*, and *ERBB2* mRNA expression as POSITIVE, NEGATIVE, INDETERMINATE, INVALID, or ERROR. Samples classified as INDETERMINATE (only applicable for *PGR* and/or *MKi67* results, where the dCt value is below the specified cutoff value and the *CYFIP1* reference gene Ct is greater than 31) or INVALID (*CYFIP1* Ct > 35) were repeated using a more concentrated lysate (i.e. 260 μl FFPE lysis reagent, 5 μl PK, and 260 μl ethanol), whereas samples classified as ERROR were repeated using the remaining sample lysate.

### Clinical evaluation of surrogate subtyping

For each surgical specimen, the IHC and STRAT4 analyses were then used to determine the surrogate breast cancer subtype (i.e. Luminal A-like, Luminal B-like (HER2- or HER2+), HER2+ (non-luminal), and Triple-negative) and recommended treatment for breast cancer patients according to national guidelines [22]. In brief, the Ki-67 cut-off was set to 20% to differentiate Luminal A-like breast cancer (Ki-67 < 20%) from Luminal B-like HER2- (Ki-67 ≥ 20%) and Luminal B-like HER2+ tumors (any Ki-67) [1]. Patients with subsequent changes in surrogate subtyping according to the STRAT4 assay results for *ESR1, PGR, ERBB2,* and *MKi67* gene expression were further evaluated by the medical and surgical oncologists (K.L. and S.J.), whom were blinded to the administered treatment for each patient.

### Statistical analyses

Statistical analyses were performed using a 0.05 *p*-value cutoff for statistical significance in MedCalc Statistical Software (version 18.10.2) or R/Bioconductor (version 3.6.0). To assess the concordance between STRAT4 and the reference method(s) (IHC or SISH, as applicable), standard 2 × 2 cross-tables were utilized along with calculation of the overall percent agreement (OPA), positive percent agreement (PPA) negative percent agreement (NPA) positive predictive value (PPV), and negative predictive value (NPV). A kappa statistic with 95% two-sided confidence intervals (CI) was calculated to estimate the overall agreement between the compared methods, with k-values > 0.8, between 0.6 and 0.8, 0.4 and 0.6, < 0.4, and <  0.2 classified as very good, good, moderate, fair, and poor agreement, respectively [23]. Scatterplots were constructed using the ggplot2 package (version 3.3.0) in R [24].

## Results

In total, the STRAT4 assay was performed twice for 12/200 samples due to INDETERMINATE Ki-67 results for four CNBs and three surgical specimens, ERROR for one CNB sample and three surgical specimens, as well as, INVALID for one surgical specimen. After repeating the STRAT4 protocol with the remaining tissue lysate or a more concentrated sample, the 12 samples yielded valid test results, i.e. POSITIVE or NEGATIVE.

### Good agreement between STRAT4 and IHC when each biomarker is investigated separately

**Fig. 1:**
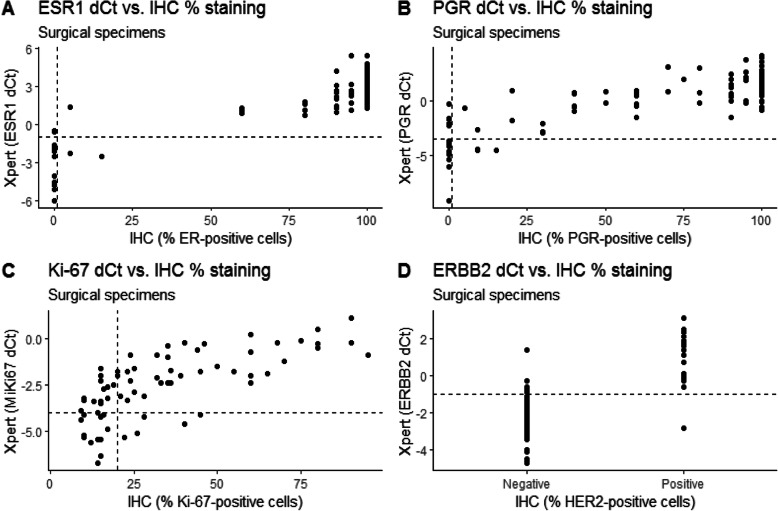
Comparison of ER, PR, Ki-67, and HER2 status according to IHC (ER, PR and Ki-67 considered to be positive with ≥1%, ≥1, and 20% immunostaining respectively, HercepTest scored 2+ (with confirmed HER2 amplification using SISH testing) or 3+ considered to be HER2-positive) and STRAT4 (ER, PR, Ki-67, and HER2 considered to be positive with dCt cut offs ≥ − 1.0, ≥ − 3.5, ≥ − 4.0 and ≥ −1.0 respectively) in the surgical specimens

**Fig. 2:**
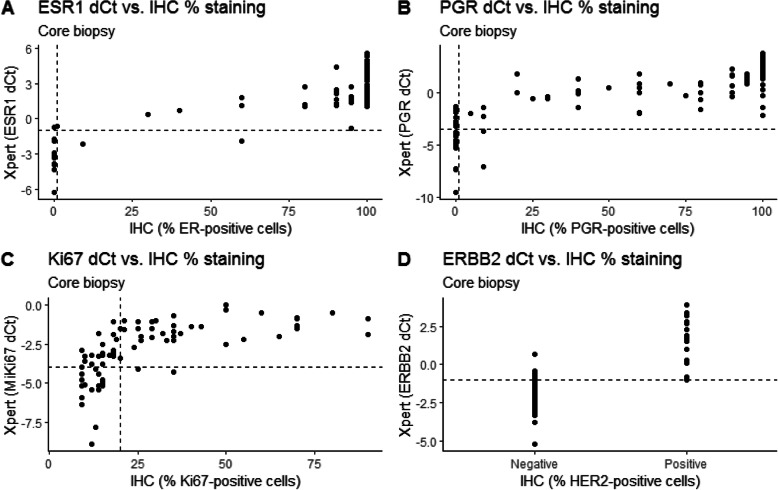
Comparison of ER, PR, Ki-67, and HER2 status according to IHC (ER, PR and Ki-67 considered to be positive with ≥1%, ≥1, and 20% immunostaining respectively, HercepTest scored 2+ (with confirmed HER2 amplification using SISH testing) or 3+ considered to be HER2-positive) and STRAT4 (ER, PR, Ki-67, and HER2 considered to be positive with dCt cut offs ≥ − 1.0, ≥ − 3.5, ≥ − 4.0 and ≥ − 1.0 respectively) in the core biopsies

The Cohen’s kappa value and the overall percent agreement (OPA) were calculated to determine the concordance between the STRAT4 and IHC results (i.e. STRAT4 vs STRAT4, and STRAT4 vs IHC) for both sample types (i.e. CNB and surgical specimen; Table 2). In general, agreement between ER status was very good (k-values > 0.8) for all comparisons, while PR had good agreement (k-values between 0.6 and 0.8), HER2 ranged from good to very good agreement, and Ki-67 had moderate agreement (k-values between 0.4 and 0.6). These findings were in line with the OPA rates, which showed the highest OPAs for ER (range, 96–99%), PR (range, 89–94%), and HER2 status (range, 86–91%). Compared to IHC, STRAT4 detected a high number of false positives for Ki-67 and HER2 in the CNB samples. ER status was found to be most consistent between the two analytical methods, while Ki-67 status was least consistent when determined using Xpert MKi67 dCt values (Figs. 1 and 2).

**Table 2:** Concordance between STRAT4 and IHC for core biopsy and surgical specimens

Comparisons	Target	True pos	True neg	False pos	False neg	OPA (%)	PPA (%)	NPA (%)	PPV (%)	NPV (%)	Kappa (95% CI^**a**^)
**STRAT4 vs IHC, both CNB**	**ER**	85	12	1	2	97.0	97.7	92.3	98.8	85.7	0.87 (0.73–1.0)
	**PR**	76	13	9	2	89.0	97.4	59.1	89.4	86.7	0.64 (0.44–0.83)
	**Ki-67**	37	39	22	2	76.0	94.9	63.9	62.7	95.1	0.54 (0.39–0.69)
	**HER2**	18	69	13	0	87.0	100.0	84.1	58.1	100.0	0.66 (0.49–0.82)
**STRAT4 vs IHC, both surgical specimen**	**ER**	85	11	2	2	96.0	97.7	84.6	97.7	84.6	0.82 (0.65–0.99)
	**PR**	78	14	5	3	92.0	96.3	73.7	94.0	82.4	0.73 (0.55–0.91)
	**Ki-67**	39	39	14	8	78.0	83.0	73.6	73.6	83.0	0.56 (0.40–0.72)
	**HER2**	17	74	8	1	91.0	94.4	90.2	68.0	98.7	0.73 (0.57–0.89)
**CNB vs surgical specimen, both STRAT4**	**ER**	86	13	0	1	99.0	99	100	100	93	0.96 (0.87–1.0)
	**PR**	81	13	4	2	94.0	98	76	95	87	0.78 (0.61–0.95)
	**Ki-67**	44	32	15	9	76.0	83	68	75	78	0.51 (0.35–0.68)
	**HER2**	21	65	10	4	86.0	84	87	68	94	0.65 (0.49–0.82)

^a^Cohen’s kappa κ-values: > 0.8 indicated very good agreement, between 0.6 and 0.8 indicated good agreement between 0.4 and 0.6 was considered as moderate agreement, < 0.4 as fair, < 0.2 as poor agreement

### Differences in concordance between two or more biomarkers assessed simultaneously

We then assessed whether two or more of the biomarkers (ER, PR, Ki-67, and HER2) were concordant for both IHC and STRAT4 (Table 3). Agreement between the biomarkers was generally lower in CNBs than surgical specimens, in particular for the four biomarkers (ER, PR, Ki-67, and HER2; 57%) and three biomarkers (ER, Ki-67, and HER2; 64%). In surgical specimens, the lowest concordance between IHC and STRAT4 was also found for the four biomarkers (ER, PR, Ki-67, and HER2; 66%). In contrast, the highest concordance between the two analytical methods were observed for ER and HER2 in surgical specimens (87%) and CNB (84%).

**Table 3:** Concordance between STRAT4 and IHC for two or more biomarkers

Comparisons	Biomarkers	OPA% (95% CI)
**STRAT4 vs IHC, both CNB**	4-ways^a^ STRAT4 vs 4-ways^a^ IHC (ER, PR, Ki-67 and HER2)	57 (46.7–66.9)
	3-ways^a^ STRAT4 vs 3-ways^a^ IHC (ER, Ki-67 and HER2)	64 (53.8–73.4)
	2-ways^a^ STRAT4 vs 2-ways^a^ IHC (ER and HER2)	84 (75.3–90.6)
	2-ways^a^ STRAT4 vs 2-ways^a^ IHC (ER and Ki-67)	74 (64.3–82.3)
**STRAT4 vs IHC, both surgical specimen**	4-ways^a^ STRAT4 vs 4-ways^a^ IHC (ER, PR, Ki-67 and HER2)	66 (55.8–75.2)
	3-ways^a^ STRAT4 vs 3-ways^a^ IHC (ER, Ki-67 and HER2)	70 (60.0–78.8)
	2-ways^a^ STRAT4 vs 2-ways^a^ IHC (ER and HER2)	87 (78.8–93.0)
	2-ways^a^ STRAT4 vs 2-ways^a^ IHC (ER and Ki-67)	75 (65.3–83.1)

^a^Agreement with respect to 4-ways analysis means total concordance with respect to all four biomarkers (ER, PR, Ki-67, and HER2), 3-ways that all three biomarkers match, etc.

### STRAT4-based subtyping may potentially result in more treatment of breast cancer patients

To evaluate the clinical utility of the STRAT4 assay, STRAT4 based biomarker assessment was first used to stratify the 100 surgical specimens into the surrogate breast cancer subtypes (Table 4). Subtyping was then used to compare administered treatment (according to IHC-based subtyping) to theoretical treatment decisions based on STRAT4 subtyping. In total, 74 (74%) specimens were concordant for the breast cancer subtypes, regardless of the method used (IHC or STRAT4). However, discordant subtyping was found to be due to changes in ER- (*n* = 5), Ki-67- (*n* = 12), and HER2-status (*n* = 9). Reevaluation of the IHC slides (by A.K. and S.J.) for the 26 discordant cases demonstrated the presence of DCIS in 4/26 cases and peritumoral normal tissue in all samples.

**Table 4:**
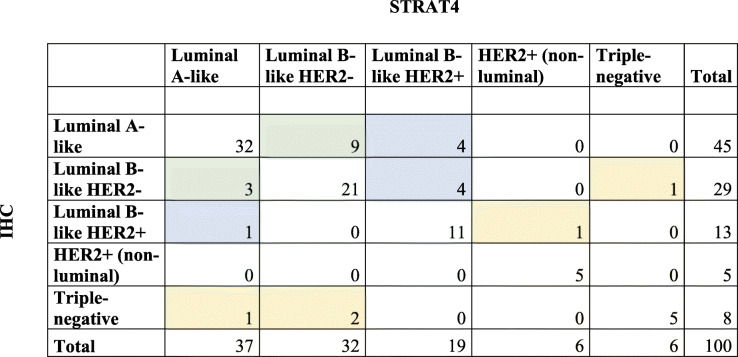
Comparison of surrogate subtype in the 100 surgical specimens determined by IHC vs STRAT4

Color depicts subtype change in surgical specimen IHC vs STRAT4 due to ER- (yellow), Ki-67- (green), and HER2 status (blue)

**Fig. 3:**
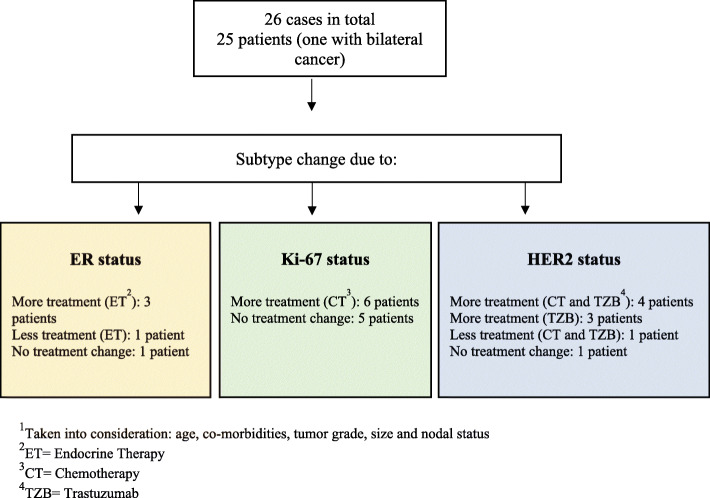
Changed treatment decisions if STRAT4 had been the gold standard^1^. ^1^Taken into consideration: age, co-morbidities, tumor grade, size and nodal status. ^2^ET = Endocrine Therapy. ^3^CT = Chemotherapy. ^4^TZB = Trastuzumab

We then conducted a review of the medical records for the 25 discordant patients (one with bilateral breast cancer) to evaluate whether changes in subtype according to STRAT4 would have resulted in changed treatment decisions (Fig. 3). For 18 of the 25 patients with subtype changes, recommendations for adjuvant treatment would have changed with additional endocrine therapy for three patients, chemotherapy for six patients, trastuzumab for three patients, and combination therapy with chemotherapy and trastuzumab for four patients. Two patients would have been recommended less treatment: one patient would have not been recommended endocrine therapy, while the other patient would have not been recommended combination therapy with chemotherapy and trastuzumab.

## Discussion

Here, we show that the concordance between STRAT4 and IHC is relatively good for ER, PR, Ki-67, and HER2, if each variable is investigated separately, which is in line with previous findings from Wu et al. [20] and Wasserman et al. [19]. However, surrogate subtype stratification (based on STRAT4 or IHC) in the surgical specimen differed for 26 cases (26%), which would potentially have changed the treatment recommendations for 18 patients (18%). The discrepancies between the STRAT4 and IHC results for the 26 cases may have, in part, been due to the presence of DCIS in four samples and peritumoral normal tissue in all the samples.

The tumor intrinsic/surrogate subtype is one of the most important factors for therapy planning, together with tumor size, grade, nodal status, age and co-morbidities that aids in choice of neoadjuvant and adjuvant treatment. To be able to provide the best individually tailored pre- and postoperative care for a breast cancer patient, the treating physician is dependent on reliable information regarding tumor biomarkers in CNBs and surgical specimens. These biomarkers need to be taken into account as a panel. The present study demonstrated fair to good concordance between STRAT4 and IHC for individual variables (ER, PR, Ki-67, and HER2), but concordance for all four variables together was less favorable.

While the pathologist solely examines neoplastic cells in IHC slides, gene assays frequently analyze RNA levels in the whole tumor mass (containing both neoplastic and non-neoplastic cells). As surrogate subtyping and, thus, treatment planning are dependent on the accurate assessment of ER, PR, Ki-67, and HER2 expression [3, 25, 26], routine diagnostic tools should be robust, reliable and reproducible. Gupta et al. suggested that macrodissection is sufficient [27], but we suggest that microdissection could potentially be useful to further improve OPA between these analytical methods. As long as an H&E slide is available, manual microdissection would require approximately 5 min of the pathologist’s time to mark the unnecessary, non-invasive areas and an additional 5–10 min for the histotechnician to perform the microdissection. The discordant cases for ER and HER2 status may have depended on the presence of adjacent ER/PR-positive normal breast tissue and/or HER2-positive ductal cancer in situ components. Reevaluation of the eight HER2 discordant cases (HER2 negative according to IHC, but HER2 positive according to STRAT4) revealed the presence of DCIS in four cases and peritumoral normal tissue in all eight cases. However, DCIS could also have been present in the corresponding FFPE section in which the STRAT4 analysis was performed. Of the eight discordant cases, seven were ductal carcinoma (including the four cases where DCIS was present) and one was mucinous cancer. Therefore, reevaluation of the IHC slides could resolve the discrepant cases and highlighted potential pitfalls with the STRAT4 assay.

In the present study, we revealed a number of Xpert *ERBB2* false positive cases, with dCt values just over the current *ERBB2* dCt cutoff (dCt = − 1.0). The current *ERBB2* cutoff was initially developed and prospectively validated in retrospective FFPE samples, some of which had aged > 10 years from the time of initial collection and block preparation. These samples may have experienced RNA degradation over time, which would have underestimated a more optimal concordance cutoff had prospectively collected samples, such as the samples used in the present study, been used in the original validation studies. Therefore, further studies are recommended to reevaluate an appropriate *ERBB2* dCt cutoff in prospectively collected specimens and using WHO guidelines (including The European Society for Medical Oncology (ESMO), American Society of Clinical Oncology (ASCO), St. Gallen International Breast Cancer Expert Panel and the National Comprehensive Cancer network (NCCN)) for defining HER2-positivity.

From a clinical point of view, use of STRAT4 instead of IHC to approximate the surrogate subtypes would theoretically have resulted in a high number patients receiving more treatment, primarily chemotherapy and trastuzumab. In patients correctly identified as HER2+, there is an overall survival benefit when given a combination of chemotherapy and HER2-targeted treatment [3]. However, chemotherapy has side effects and the administration of adjuvant chemotherapy involves careful consideration. Overtreatment with chemotherapy and trastuzumab will lead to unnecessary suffering with potentially irreversible side effects and no additional benefit [28, 29]. For a large group of ER-positive breast cancer patients, endocrine treatment can also be difficult to tolerate. Recent studies have shown that side effects can have an impact on decisions to discontinue therapy early [30, 31]. Furthermore, the significant financial burden of cancer care can also be a driving factor for therapy choice, particularly in countries that in part or fully subsidize treatment costs. It is, therefore, important that the most suitable treatment is recommended [32–34]. Further validation studies, ideally conducted using prospectively collected specimens and potentially alternate dCt cutoffs (particularly for *ERBB2* and *MKi67* given the lack of consensus on the IHC cutoffs for Ki-67), are warranted to demonstrate improved STRAT4 accuracy and ensure a correct and robust assessment of all biomarkers. In addition, the clinical utility of STRAT4 should be compared with established multigene prognostic tests for breast cancer (e.g. PAM50/Prosigna, Mammaprint/BluePrint).

## Conclusions

In summary, use of other diagnostic methods to assess surrogate BC subtyping in the surgical specimen (e.g. STRAT4) may theoretically result in patients receiving more adjuvant treatment, primarily with chemotherapy and trastuzumab. It is imperative that ER, PR, Ki67, and HER2 biomarker assessment in CNBs and surgical specimens is accurate when new diagnostic methods are tested, as the results may have an impact on treatment decisions. Inadequate treatment can have serious consequences for the patient with regards to side effects and survival. Furthermore, comparison with established multigene prognostic tests for breast cancer should also be assessed so that the accuracy of the biomarkers is more precise.

## Data Availability

The data that support the findings of this study are available from Cepheid but restrictions apply to the availability of these data, which were used under license for the current study, and so are not publicly available. Data are however available from the authors upon reasonable request and with permission of Cepheid. The datasets used and analyzed during the current study are available from the corresponding author on reasonable request.

## Abbreviations

BC: Breast cancer
ER: Estrogen
PR: Progesterone
IHC: Immunohistochemistry
CNB: Core needle biopsy
STRAT4: Xpert® Breast Cancer STRAT4 Assay
FFPE: Formalin-fixed paraffin-embedded
NBF: Neutral Buffered Formalin
H&E: Hematoxylin and eosin
Ct: Cycle threshold
dCt: Delta cycle threshold
